# Primary liver carcinoid tumour with a Zollinger Ellison syndrome - an unusual diagnosis: a case report

**DOI:** 10.4076/1757-1626-2-6346

**Published:** 2009-08-07

**Authors:** Gabriela Rascarachi, Mónica Sierra, Mercedes Hernando, Rubén Diez, Laura Arias, Francisco Jorquera, Santiago Vivas, José Luis Olcoz

**Affiliations:** 1 Gastroenterology Unit, Complejo Hospitalario Leónc/Altos de Nava s/n, 24071, LeónSpain; 2 Pathology Department, Complejo Hospitalario Leónc/Altos de Nava s/n, 24071, LeónSpain; 3 Institute of Biomedicine, University of LeónCampus Universitario, 24071, LeónSpain

## Abstract

Carcinoids are neuroendocrine tumours which may secrete hormones like gastrin, insulin, ACTH, etc. Liver is a common site for metastasis of carcinoid origin and an unusual site for a primary carcinoid tumour to arise.

We present the case of a 51-year-old Caucasian man with diarrhoea, weight loss, duodenum ulcers and a liver mass in ultrasonography. A primary hepatic carcinoid tumour with a Zollinger Ellison syndrome was diagnosed. Surgery resection was performed and the patient remained free of symptoms two years after, with normalisation of gastrin levels.

Primary hepatic carcinoid tumour represents an uncommon diagnosis, based on radiological and pathological features. The exclusion of different primary locations is necessary. Once associated with a Zollinger Ellison syndrome, diagnose may be more complicated and challenging since only 7 cases of hepatic carcinoids with gastrin secretion were reported in medical literature.

A review of medical literature is performed and diagnoses tools that should be used for an accurate diagnosis and available treatment approaches are commented here.

## Introduction

Gastrin is predominantly produced by G cells of the gastric antrum and plays two major roles in the gastrointestinal physiology: (i) stimulation of gastric acid secretion and (ii) secondly as a trophic hormone for epithelial and enterochromaffin cells growth [1,2]. There are many causes of chronic gastrinemia and most of them are associated with gastrin secreting tumours (gastrinomas and gastrin secreting carcinoid tumours). Other causes are relationated with high continuous antral pH (chronic atrophic gastritis, truncal vagotomy without antrectomy, chronic antisecretory treatment, etc). Gastrinomas are usually associated with the Zollinger Ellison syndrome (ZES) which is characterized by multiple peptic ulcers (most in the first portion of duodenum) and severe diarrhoea with inflammatory, osmotic or malabsorptive features due to the excessive acidification of the duodenum and the lower small bowel [2].

Carcinoid tumours are neoplasms derived from the neuroendocrine system and usually secret hormones like gastrin, chromogranin A, serotonin, ACTH, insulin, etc. [3]. The majority arises within the gastrointestinal tract, mostly in the appendix and terminal ileum [3], few within other organs like bronchi. Liver is a common site for metastasis of carcinoid origin and an unusual site for a primary carcinoid tumour to arise.

## Case presentation

A 51-year-old Caucasian man of Spanish nationality was admitted to our department for diarrhoea. The symptoms began 11 years ago and were intermittent and mostly nocturnal. He worsened 3 months before admission with exacerbation of number of deposition, abdominal pain and weight loss. He was on PPI therapy on demand and at high doses for pyrosis. He consumed no toxics. His medical history was irrelevant and his physical examination was unremarkable except for the presence of a mass in his left upper quadrant of abdomen. No palpable lymph nodes and no splenomegaly were found.

A complete blood count revealed mild leukocytosis (leukocytes 16.000, neutrophils 83%). Coagulation study and biochemistry tests including liver, kidney and thyroid gland function, iron metabolism and tumour markers presented no remarkable alterations. The reactive C protein was of 42.7 mg/dl (normal: 0-5 mg/dl). Gastrin level was of 114 ng/ml (normal: 13-115 ng/ml). Secretin induced test at 15 min was of 322 ng/ml. VIP and 5-HIAA levels were normal. The stool exam was negative for bacterial and parasites.

**Figure 1.**
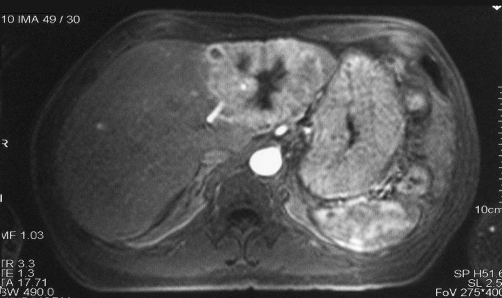
Axial section of MRI showing a left lobe liver mass with central necrosis and contrast uptake.

Abdominal ultrasound revealed a liver mass in the 2^nd^ and 3^rd^ segment of a 10 x 8 cm diameter and small bowel distension. A helical computed tomography scan and a magnetic resonance imaging confirmed the liver mass with contrast uptake (Figure 1). The fine-needle aspiration of the liver mass was informed as metastasis of an adenocarcinoma.

Upper endoscopy was performed and showed grade B (Los Angeles classification) esophagitis and gastritis as well as hypertrophy of the gastric folds and multiple duodenum ulcers with unspecific biopsy and H. Pylori negative. Barium intestinal study showed the same findings, without any small bowel lesions.

The gammagraphy with 111 In-DTPA octreotide showed pathologic uptake in the upper abdomen, especially at the jejunum wall, with high expression of serotonin receptors. A PET study was performed which showed high abnormal uptake in the liver mass as well as a diffuse uptake into the axils, small bowel wall and spleen. The study was completed by endoscopic capsule examination which did not reveal alterations at that level and with a complete colonoscopy without relevant findings. The echocardiography was normal.

**Figure 2.**
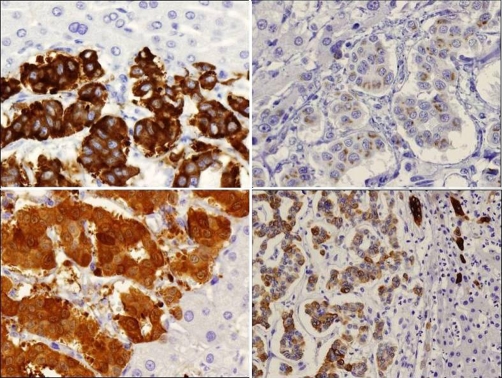
Immunohistochemical staining of the primary hepatic carcinoid tumour specimen. Positive staining for synaptophysin **(a)**, chromogranin **(b)**, enolase **(c)** and cytokeratin 7 **(d)** in tumour sample and negative in normal liver parenchyma (synaptophysin, chromogranin, *enolase* × 400, cytokeratin 7 × 200).

**Figure 3.**
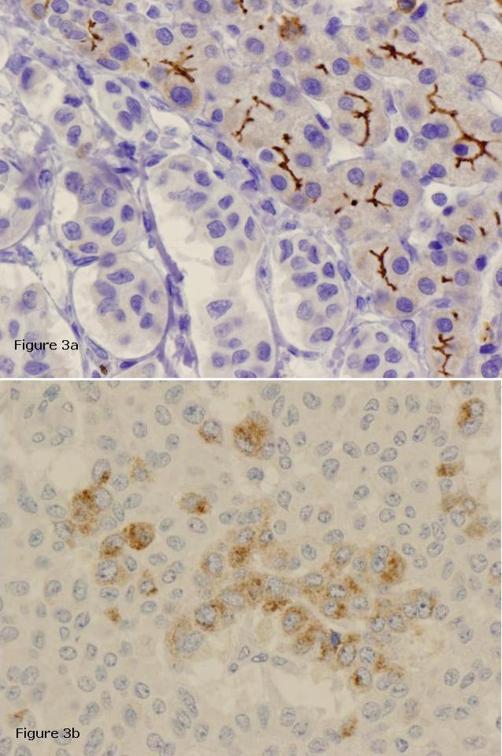
Immunohistochemical staining of the primary hepatic carcinoid tumour specimen. Panel **(a)** negative CEA staining in tumour sample and positive in normal liver parenchyma (CEA, × 200). Panel **(b)** moderate gastrin staining in tumour sample (gastrin, × 400).

A left hepatectomy was performed and the pathologic diagnosis pointed out to a primary neuroendocrine tumour (carcinoid tumour) with poor mitotic activity and important necrosis. Inmunohistochemistry, on paraffin-embedded formalin fixed sections of the tumour, showed positive staining for synaptophysin, chromogranine, enolase, CK7 (Figure. 2a, 2b, 2c , 2d) and negative staining for CEA (Figure 3A). A moderate staining was detected for gastrin (Figure 3B).

During the follow up, the patient referred total disappearance of the initial clinical symptoms. Upper endoscopy revealed no duodenum ulcers and his serum gastrin levels decreased till 19 pg/ml two months after surgery.

The patient remained free of symptoms two years after surgery.

## Discussion

We report here a case of a primary hepatic carcinoid tumour (PHCT) associated with a Zollinger Ellison syndrome. The primary hepatic carcinoid tumours are uncommon [3] and represent an intriguing diagnosis to solve. The demonstration of the hepatic origin of a neuroendocrine tumour is difficult since the liver is a frequent site for metastases of different types of tumours: intestinal carcinoids, primary tumours (hepatocarcinoma, etc) and benign pathology (nodular focal hyperplasia, etc).

Carcinoid tumours have their origin in neuroendocrine stem cells (known as enterochromaffin cells or APUD cells (Amine Precursor Uptake Decarboxilase) derived from the embrionary neuronal crest. Cells that migrated from the neuronal crest can give rise to gastrointestinal carcinoid tumours to foregut, midgut and hindgut, being the most common localization the appendix and the small bowel [4]. Liver has few or none enterochromaffin cells, unlike other organs (pancreas, for example).

Some theories for the origin of PHCTs have been proposed. The most accepted thories are: (i) ectopic cells of pancreatic or adrenal tissue, (ii) neuroectodermal origin cells, (iii) neuroendocrine programmed ectoblasts [5]. It is believed that, during the early stages of regeneration, the bile ducts epithelium displays neuroendocrine characteristics and cytoplasmatic chromogranin A expression, due to chronic inflammation [6].

In general, PHCT may vary in size, ranging from 1 cm to 20 cm [7] and their aspect is of a unique mass [3] completely separated from the surrounding liver tissue. Females tend to be affected more often than males (1.4:1) and the age of presentation may range between 18 to 84 years, with an average of 50 years [3,8].

The clinical presentation is atypical, with symptoms like upper abdominal pain or discomfort like fullness, as well as diarrhoea or weight loss [3]. Only a small percent (7%-13%) of patients accuses symptoms of a typical carcinoid syndrome [3,7].

The radiology diagnosis has poor specificity as the appearance on ultrasound, CT or MRI is of a solid mass with cystic areas and hyperechoic or mixed pattern, with central or peripheral calcifications and fibrous scars. The majority are intense, hypervascularised tumours, which explains the contrast enhancement in ultrasound and CT images, in a similar fashion as the hepatocarcinoma. [9]. When diagnosing a neuroendocrine tumour, an octreoscan is always recommended since it may detect small metastasic deposits. PET scan may visualize somatostatin receptor type 2 lacking neuroendocrine tumours which are impossible to detect on somatostatin receptor scintigraphy.

The gold standard for diagnosis is the liver biopsy since it may differentiate the lesion from other types of tumours. Cautions must be taken in order to avoid complications like carcinoid crisis as a recent case report shows [10]. From the histologic point of view the hepatic carcinoma appears as a hemorrhagic, not capsulated mass, with central, irregular fibrosis and hyaline degeneration. The tumoural cells display an eosinophilic cytoplasm and irregular, hyperchromic nucleus. In inmunohistochemistry these cells present a strong positivity for neurosecretory markers as chromogranin, synaptophysin, neuron specific enolase meanwhile markers as serotonin, pancreatic polypeptide or gastrin are inconsistently positive [8].

The treatment is the surgical resection, when possible. Almost 85% of primary hepatic carcinoid tumour patients may have a resectable disease [11,12]. Survival and recurrence tares five years after surgery are of 80-92.5% [8,13] and 18% respectively [14]. When a not well defined lesion is observed, a palliative cyto-reductive surgery in combination with transcatheter arterial embolization (TACE) and subsequent administration of lanreotide (long acting somatostatin analogue) might be effective, as Touloumis et al. showed recently [12].

In our patient’s case, the immunohistochemistry result corresponded to a type A carcinoid tumour (APUDOMA). The moderate staining for gastrin pointed to gastrin secreting PHCT, associated thus with a Zollinger Ellison syndrome. In a recent review, gastrin was the most common hormone secreted of all cases (7/69) of primary hepatic carcinoid published till date [3].

Primary hepatic carcinoid tumours are rare entities that sometimes may be associated with syndromes like ZES, carcinoid, etc. induced by neuroendocrine hormones secretion (gastrin, 5-HIAA, ACTH; etc). An early and pertinent diagnosis offers the possibility of surgery resection, with a good 5 early survival rate and a poor relapse rate.

## Authors’ information

Francisco Jorquera and Jose Luis Olcoz are members of CIBEREHD group, founded by Instituto Carlos III, Spain.

## Abbreviations

APUD: Amine Precursor Uptake Decarboxilase
CEA: carcinoembrionary antigen
CK7: cytokeratin 7
CT: computer tomography
PET: positone emission tomography
PHCT: primary hepatic carcinoid tumour
PPI: proton pomp inhibitor
TACE: transcatheter arterial embolization
ZES: Zollinger Ellison syndrome
